# Synergistically Promoting Bone Regeneration by Icariin-Incorporated Porous Microcarriers and Decellularized Extracellular Matrix Derived From Bone Marrow Mesenchymal Stem Cells

**DOI:** 10.3389/fbioe.2022.824025

**Published:** 2022-04-07

**Authors:** Mengyang Zhou, Min Guo, Xincui Shi, Jie Ma, Shutao Wang, Shuo Wu, Weiqun Yan, Feng Wu, Peibiao Zhang

**Affiliations:** ^1^ School of Pharmaceutical Sciences, Jilin University, Changchun, China; ^2^ Key Laboratory of Polymer Ecomaterials, Changchun Institute of Applied Chemistry, Chinese Academy of Sciences, Changchun, China; ^3^ Foshan Hospital of Traditional Chinese Medicine/Foshan Hospital of TCM, Foshan, China

**Keywords:** poly(glycolide-co-caprolactone) (PGCL), icariin (ICA), porous microcarriers, decellularized extracellular matrix (dECM), bone regeneration, bone marrow mesenchymal stem cell (BMSC)

## Abstract

Multifunctionality has becoming essential for bone tissue engineering materials, such as drug release. In this study, icariin (ICA)-incorporated poly(glycolide-*co*-caprolactone) (PGCL) porous microcarriers were fabricated and then coated with decellularized extracellular matrix (dECM) which was derived from bone marrow mesenchymal stem cells (BMSC). The porous structure was generated due to the soluble gelatin within the microcarriers. The initial released ICA in microcarriers regulated osteogenic ECM production by BMSCs during ECM formation. The dECM could further synergistically enhance the migration and osteogenic differentiation of BMSCs together with ICA as indicated by the transwell migration assay, ALP and ARS staining, as well as gene and protein expression. Furthermore, *in vivo* results also showed that dECM and ICA exhibited excellent synergistic effects in repairing rat calvarial defects. These findings suggest that the porous microcarriers loaded with ICA and dECM coatings have great potential in the field of bone tissue engineering.

## Introduction

Large bone defects caused by trauma or disease have been a major problem in orthopaedics due to their limited regenerative capacity. Specifically, cranial defects are one of the most challenging issues due to their defective characteristics and local environment (Tang et al., 2016; Roseti et al., 2017; Charbonnier et al., 2019). Although autologous bone grafting is regarded as the “gold standard” for the treatment of bone defects, it has many limitations such as insufficient donor sources, secondary injury and infection risks (Turnbull et al., 2018; Nie et al., 2020). Therefore, the design and development of new bone substitutes are urgently needed in the experimental research and clinical orthopaedic applicatiuons (Karadjian et al., 2019). The ideal bone substitutes should have both osteoconductivity and osteoinductivity properties (Datta et al., 2006). Biocompatibility, biodegradability and mechanical properties are also the basic requirements in addition to the functional properties of drug loading and environmental responsiveness. They should also be capable of supporting cellular adhesion, growth and the formation of extracellular matrix (Agarwal et al., 2008; Kelleher and Vacanti, 2010; Guo et al., 2012; Grande Tovar et al., 2019).

Recently, microcarriers have been received considerable attention in the field of tissue engineering as a new type of spherical scaffold with biological functions. Composite microcarriers have been used for the repair of bone defects (Jang et al., 2020; Peticone et al., 2020; Li et al., 2021). Compared to other types of scaffolds, microcarriers can provide a highly specific surface area for cell growth, maintaining a differentiated cell phenotype. Furthermore, they can be used as injectable materials either loading with cells or not. The highly interconnected structure of porous microcarriers facilitates the transfer of oxygen and nutrients and provides more space for cell growth (Malda and Frondoza, 2006; Gao et al., 2017; Zhou Z. et al., 2020). Poly (glycolide-co-caprolactone) (PGCL) is a co-polymer synthesized from glycolide and caprolactone monomer with stannous octoate as the catalyst. The polymer displays good elastic mechanical properties and biodegradability. It has been reported that PGCL scaffolds are much more elastic than the poly (lactic-co-glycolic acid) (PLGA) scaffolds made by the same methed (Kwon et al., 2001; Lee et al., 2003; Piao et al., 2007; Sharma et al., 2018). Hydroxyapatite (HA) is the major component of bone minerals and has been widely used in bone tissue engineering scaffolds due to its excellent biocompatibility, biodegradability and osteoconductivity (Barrère et al., 2006; Kim et al., 2017; Yu et al., 2017). Gelatin is a natural biopolymer that is readily soluble under physiological conditions. It has a variety of bioactive sequences and structural fragments that enhance cell function and promote the formation of the extracellular matrix (ECM), and is now widely used in tissue engineering and biomaterials. In addition, gelatine has the advantages of promoting cell adhesion, having good biocompatibility, and being cost-effective (Ranganathan et al., 2019; Han et al., 2021; Raj Preeth et al., 2021). On the other hand, gelatin has been used as a typical porogen for preparing microcarriers by the emulsion-solidification technique (Zhou Z. et al., 2020). Icariin (ICA), the main active ingredient of Epimedium, is a flavonoid phytomolecule that can promote osteoblast differentiation and inhibit osteoclast formation (Lai et al., 2018; Ma et al., 2019; Wang et al., 2019; Xie et al., 2019; Zhang et al., 2019). It has been reported that ICA could increase cartilage ECM synthesis and inhibit ECM degradation (Liu et al., 2010; Zhang et al., 2012). Moreover, it has been found that ICA enhanced bone marrow mesenchymal stem cell (BMSC) osteogenic differentiation and promoted matrix calcification (Xu et al., 2018; Yao et al., 2019a; Xie et al., 2019). ICA is also more stable and less expensive than exogenous growth factors (Yang et al., 2018; Liu et al., 2019). Therefore, the incorporation of ICA within the bioactive scaffolds will provide us an attractive biofunctional platform for bone tissue engineering.

ECM is mainly consisted of collagen, proteoglycans, glycoproteins and a certain amount of growth factors and other signaling molecules (Kang et al., 2011; Kim et al., 2015). These growth factors can be released with the degradation of ECM and thereby regulate tissue regeneration by modulating cell functions (Gaffney et al., 2017). In addition, it has been reported that ECM could also recruit endogenous progenitors and stem cells to the injury site and direct the self-healing progress of lesions. It is widely recognized that ECM has a crucial influence on cell proliferation and differentiation during tissue reconstruction and organogenesis (Wang et al., 2020). In recent years, combining ECM with biodegradable materials has received considerable attention. To avoid from triggering an immune response when implanted *in vivo*, ECM-modified synthetic materials were generated by decellularization (Gaffney et al., 2017; Kim et al., 2019; Lee et al., 2020). Compared to animal tissues, cell-derived ECM has the advantages of avoiding pathogen transfer and immune response. BMSCs with the character of high proliferation and osteogenic capacity, are the predominant MSCs used in tissue engineering. (Liu et al., 2020). dECM derived from BMSCs has been reported for promoting osteogenic differentiation and bone matrix deposition (Gao et al., 2018; Zhou S. et al., 2020). In addition, Datta et al. have found that titanium mesh discs containing osteogenic ECM significantly enhance the deposition of bone matrix (Datta et al., 2006).

In this study, porous PGCL/HA microcarriers loaded with ICA were fabricated using gelatin as porogens. Gelatin can help the microcarriers increase cell adhesion and at the same time prevent burst loss of drugs through *in situ* soluble and pore formation in physiological environments. Subsequently, ECM was successfully deposited on the surface of the porous microcarriers by decellularization to further promote the migration and osteogenic differentiation of rat BMSCs *in vitro*. In addition, *in vivo* tests were performed to evaluate the bone repair ability of the drug-loaded and ECM encapsulated microcarriers.

## Materials and Methods

### Materials

ICA and gelatin were purchased from Aladdin (Shanghai, China). caprolactone and PVA-117 were purchased from Macklin (Shanghai, China). Glycolide (GA) was obtained from Purac (Gorinchem, Netherlands). Stannous octoate (Sn(Oct)_2_) was supplied from Sigma (St. Louis, United States).

### Synthesis of Poly(Glycolide-Co-Caprolactone)

PGCL was synthesized according to previously reported methods (Sharma et al., 2018). Briefly, a catalyst stannous octoate [Sn(Oct)_2_] (0.71 ml) diluted in toluene (2.5 ml), initiator pentaerythritol (75 mg), and caprolactone (1.2 ml) were added to the ampoule. The ampoule was heated to 90°C and kept for 4 h to form homogeneous catalysts. A round bottom flask with glycolide (11.0 g) and caprolactone (99.0 g) was heated to 90°C under reduced pressure and kept for 4 h, and then the catalyst was added to remove toluene. The reaction proceeded at 120°C for 48 h under a nitrogen atmosphere. The copolymer was dissolved in CHCl_3_, precipitated by ethanol and dried. A gel permeation chromatograph (GPC) using poly (methyl methacrylate) as a standard was acquired to measure the number average molecular weight (Mn), weight average molecular weight (Mw), and polydispersity index (PDI) of the copolymer (Sharma et al., 2018).

### Synthesis of Hydroxyapatite

Hydroxyapatite (HA) was prepared following our published methods (Zhang et al., 2015). Briefly, 5.48 g of K_2_HPO_4_·3H_2_O was dissolved in 100 ml de-ionized water and the pH was adjusted to 12 with 1 M NaOH. Then, 4.44 g of CaCl_2_ was dissolved in 60 ml deionized water. The CaCl_2_ solution was dropwisely added to the K_2_HPO_4_·3H_2_O solution with constant stirring and refluxed at 120°C for 24 h. Then it was centrifuged at 10,000 rpm for 5 min, washed with ethanol and deionized water, dried in an oven for 48 h, and ground to powder.

### Fabrication of Microcarriers

The microcarriers were fabricated by emulsion-solidification technique according to the previous literature with some modifications (Gao et al., 2017). First, HA powder was suspended in chloroform and added in a PGCL/chloroform (5%, w/v) solution and then followed by adding a gelatin solution (6.5%, w/v). The mass ratio of PGCL: HA was 9:1 (w/w), and the volume ratio of PGCL and gelatin solution was 10:1. Then the mixed solution was stirred and emulsified with a homogenizer for 3 min. Then, the water-in-oil was poured into aqueous polyvinyl alcohol (PVA) solution (3%, w/v) and stirred at 1,200 rpm overnight. The icariin-loaded microcarriers were fabricated by adding the ICA powder to the mixed solution before emulsification. The mass ratio of PGCL to HA to icariin was 90:10:0.32. In addition to this, all other steps were similar to the above, so the PGCL/HA/Gelatin microcarriers and PGCL/HA/Gelatin/ICA microcarriers were correspondingly labeled as MCs and M-ICA. Stereomicroscope and scanning electron microscopy (SEM) were used to observe the surface morphology of the two different microcarriers.

### Icariin Release

The release behavior of ICA was investigated by immersing the microcarriers in 5 ml PBS at 37°C. After 1, 3, 5, 7, 14, 21, 28 and 35 days, the PBS solution was collected and supplemented with the same volume of fresh PBS. The collected solution was extracted thrice with equal volumes of ethyl acetate and then evaporated using a rotary evaporator and redissolved in equal volumes of methanol. The ICA concentration was detected by UV Spectrophotometer at 270 nm and calculated according to the standard curve (Lai et al., 2018; Hu et al., 2020).

### Bio-Modification by Extracellular Matrix Coatings

#### Isolation and Culture of Bone Marrow Mesenchymal Stem Cells

The whole bone marrow adherent method was used for isolating BMSCs (Chi et al., 2020). Five 4-week-old male SD rats were used in the experiment. Bilateral femurs were removed 30 min after the rats death from 2% sodium pentobarbital anesthetic overdose under sterile conditions. All experiments were conducted under the ethical regulations. The bone marrow cavity was irrigated with Dulbecco’s modified Eagle’s medium-F12 (DMEM-F12; HyClone, United States) supplemented with 10% fetal bovine serum (FBS; Biological Industries, United States), and 1% penicillin and streptomycin (Hyclone, United States). After centrifugation (1,200 rpm, 10 min), the cells were resuspended in the same medium and plated in a 10 cm cell culture dish at 37°C with 5% CO_2_ and the medium was changed every 3 days. At 90% confluence, the cells were subcultured. Passage 3 BMSCs were used for identification and further experiments. The morphological characteristics and identification of BMSCs were shown in Supplementary Figure S1.

#### Morphology and Adhesion of Bone Marrow Mesenchymal Stem Cells on M-ICA Microcarriers

Rhodamine-phalloidin/DAPI staining was used to characterize cell morphology and adhesion on the M-ICA microcarriers. (Yuan et al., 2021). BMSCs (5 × 10^4^ cells/well) were seeded onto the M-ICA microcarriers (2mg/well) in 48-well plates. After culturing for 1, 3, and 7 days, the cells cultured on the M-ICA microcarriers were fixed using 4% paraformaldehyde (PFA) for 30 min and then washed three times with PBS. Subsequently, filamentous actin (F-actin) was stained with rhodamine-phalloidin (Sigma, United StatesA\) for 30 min at 37°C and cell nuclei were dyed with DAPI for 1 min at room temperature. After washing three times with PBS, images were captured with a fluorescence inverted microscope (TE 2000U, Nikon).

#### Decellularization and Decellularized ECM Morphology

The decellularization method was performed according to the literature.(Deng et al., 2020). After 7 days, the cell-cultured M-ICA microcarriers were rinsed with PBS and collected into centrifuge tubes. Then the samples were frozen at −80°C for 10 min, thawed in a constant temperature shaker at 60 rpm for 20 min at 37°C, rinsed with PBS, frozen at −80°C overnight, and freeze-dried to prepare decellularized ECM (dECM) coating on the surface of microcarriers. Such microcarriers we labeled as M-ICA@ECM. Then, scanning electron microscope (SEM) was used to observe the cellular morphology on the microcarriers.

### Osteogenic Extracellular Matrix Evaluation

#### Alkaline Phosphate Activity Assay

ALP quantification and staining were used to characterize the ALP activity of ECM-secreting BMSCs after 7 days of incubation (Yan et al., 2021). ALP quantification was measured according to the instructions of the ALP assay kit (Beyotime, China). BMSCs were seeded at 5 × 10^4^ cells/well on the MCs (2 mg/well) and M-ICA (2 mg/well) in 48-well plates. After incubation for 7 days, the samples were rinsed with PBS and lysed by adding 200 μl cell lysis buffer for Western and IP without inhibitors (Beyotime, China). The mixed lysing solutions were frozen at −80°C for 30 min, thawed at 37°C and centrifuged for 15 min at 4°C, 13,000 rpm. The *p*-nitrophenol phosphate (pNPP) assay and bicinchoninic acid (BCA) solution were added and incubated at 37°C for 30 min away from light. The OD405/OD562 ratio was read as the ALP quantitative evaluation. For ALP staining, the cell-cultured microcarriers were fixed by 4% PFA for 20 min and rinsed three times with PBS. Then ALP staining assay was performed according to the ALP staining kit (Beyotime, China) protocol. After 24 hof incubation away from light, the samples were observed with a stereomicroscope.

#### Alizarin Red Staining

To evaluate the mineralization of BMSCs culture on the different microcarriers, ARS was performed according to our published literature with some modification.(Yan et al., 2019). After culturing on the MCs and M-ICA for 7 days, BMSCs were rinsed with PBS and fixed with 4% PFA. The fixed cells were washed with PBS for 3 times, and incubated with ARS solution (Cyagen, China) for 5 min at room temperature. Followed by washing twice, the mineral deposition was observed by a stereomicroscope. Finally, cetylpyridinium chloride (CPC) was used to measure the quantification of calcium. Briefly, the samples were rinsed with deionized water and treated with 1 ml 10% CPC solution for 1 h at 37°C to desorb calcium ions. The absorbance values at 540 nm were read using a multifunctional microplate scanner (Tecan Infinite M200).

#### Quantitative Real-Time Polymerase Chain Reaction

To evaluate the effect of ICA on the ECM formation of BMSCs on the microcarriers, we detected the expression levels of osteogenic-related genes (OSX, OPN, BMP-2, BMP-4) in BMSCs by qRT-PCR (Yan et al., 2021). BMSCs were incubated on the MCs and M-ICA for 7 days, then the total RNA was extracted by Trizol Reagent (Invitrogen, United States) according to the manufacturer’s protocol and quantified by Nanodrop Plates (Infinite M200, TECAN). The cDNA was synthesized using the Prime Script RT Reagent Kit with gDNA Eraser RR047A (TaKaRa, Japan) according to the recommendations of the manufacturer. Gene expression was quantified using SYBR Premix Ex Taq RR420A (TaKaRa, Japan) and normalized to the value of GAPDH. The gene expression value was calculated with the formula: △Ct = Ct (target gene)—Ct (reference gene), and the relative = 2^−△△Ct^. The primer sequences of the relative genes are listed in Table 1.

**TABLE 1:** Primers used in quantitative real-time polymerase chain reaction (qRT-PCR).

Gene	Forward primer sequence (5′-3′)	Reverse primer sequence (5′-3′)
BMP-2	AGA​AAG​GCA​ACA​GAA​GCC​CA	ACC​ATG​GTC​GAC​CTT​TAG​GAG
BMP-4	GGA​GGA​AGA​AGA​GCA​GAG​CC	TGT​TCT​CCA​GAT​GTT​CTT​CGT​GA
OSX	GCC​AGT​AAT​CTT​CGT​GCC​AG	TAG​TGA​GCT​TCT​TCC​TGG​GGA
OPN	CCA​GCC​AAG​GAC​CAA​CTA​CA	AGT​GTT​TGC​TGT​AAT​GCG​CC
Col-Ⅰ	CCC​AGC​GGT​GGT​TAT​GAC​TT	TCG​ATC​CAG​TAC​TCT​CCG​CT
GAPDH	CTT​GTG​CAG​TGC​CAG​CCT​C	GAT​GGT​GAT​GGG​TTT​CCC​GT

### Transwell Migration Assay

To evaluate the cell migration and recruitment capacity of M-ICA@ECM, a 24-well chamber (pore size: 8 μm; Corning, United States) was used according to the product instruction (Yao et al., 2019b). After hydration, 100 μl of cell suspension (3 × 10^5^ cells/ml) without serum was placed in the upper compartment, and different microcarriers (MCs, M-ICA, M-ICA@ECM) were added into the lower compartment with 600 μl complete medium. After 12 h of incubation at 37°C, the upper surface of the transwell membrane was scraped with a cotton swab to remove the adherent cells and debris. Then, the chambers were fixed with 4% PFA for 20 min at room temperature, rinsed in PBS and stained with crystal violet for 15 min in the dark. The migrated cells were observed under a microscope.

### *In vitro* Osteogenic Differentiation of Bone Marrow Mesenchymal Stem Cells on M-ICA@ECM Microcarriers

#### Alkaline Phosphate Activity Assay and Alizarin Red Staining

To evaluate the effect of ICA incorporation and dECM coating on the ALP activity and mineralization of BMSCs, ALP assay and ARS were performed. The BMSCs were separately seeded on the MCs, M-ICA and M-ICA@ECM for 14 days, then ALP quantification, ALP staining and ARS were performed as described above.

#### qRT-PCR

To further confirm the effect of ICA incorporation and dECM coating on the osteogenic differentiation of BMSC *in vitro*, the expression of osteogenesis-related gene osteopontin (OPN) and collagen type I (Col-I) was detected by qRT-PCR after 14 days of culture.

#### Immunofluorescence Staining

The expression of osteogenesis-related protein OPN and Col-I was detected by immunofluorescence staining.(Yao et al., 2019b). The BMSCs were cultured on the various microcarriers for 14 days, fixed with 4% PFA for 20 min at room temperature and then washed with PBS. The fixed cells were permeabilized with 0.25% Triton X-100 in PBS for 10 min and blocked with 5% bovine serum albumin (BSA) in PBST (0.25% Tween-20 in PBS). Next, primary anti-Col-I (1:200, AF7001, Affinity, United States) or anti-OPN (1:200, AF0227, Affinity, United States) was incubated overnight at 4°C on a shaking table. After being washed three times with PBS, the cells were exposed to the secondary antibody, goat anti-rabbit IgG (1:1,000, ab150077 Alexa Fluor^®^488, Abcam, United Kingdom) for 2 h at room temperature in dark. Finally, 4′,6-diamidino-2-phenylindole (DAPI; Sigma, United States) was used to stain the cell nuclei. Photos were taken on a laser confocal microscope (FV1000, Olympus, Tokyo, Japan).

### *In vivo* Studies

#### Animal Models

Twenty Wistar rats were used *in vivo* experiments. After adaptive feeding for 1 week, all the animals were intraperitoneal injection of 2% sodium pentobarbital to induce general anesthesia, then two 5-mm diameter full-thickness calvarial defects were created and filled with different microcarriers. Penicillin was used to prevent infection for 1 week after surgery. All animals were randomly divided into four groups (*n* = 5): untreated (blank), implanted with MCs, M-ICA, M-ICA@ECM, respectively. Four weeks and 8 weeks after surgery, the animals were sacrificed by an overdose of anesthesia, and the skull was obtained and fixed with 4% PFA for further analysis. All animal experiments were approved by the Institutional Animal Care and Use Committee of School of Pharmaceutical Science, Jilin University.

#### Micro-CT Analysis

The calvarial bones were scanned using micro-CT (Skyscan 1,172, Bruker micro-CT, Germany) at 80 kV, 100 μA and a 0.5 mm aluminium filter. NRecon software was used to optimize scanned images, and CTvox software was used for 3D reconstruction. Afterwards, CTAn software was used to calculate the ratio of bone volume to tissue volume (BV/TV), trabecular thickness (Tb.Th), trabecular number (Tb.N), and trabecular separation (Tb.Sp).

#### Histological Observation

**FIGURE 1:**
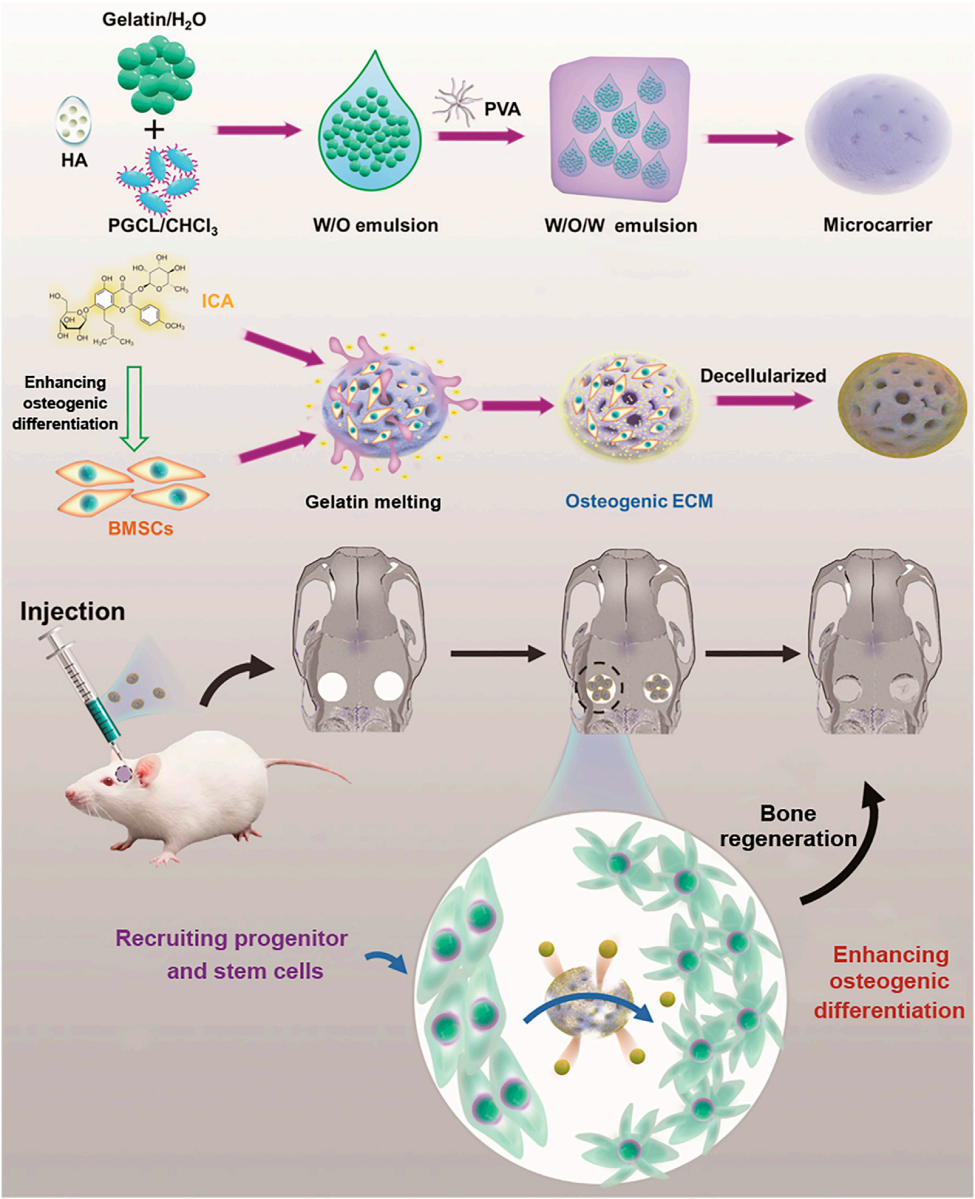
Schematic diagram of ICA-loaded microcarriers with ECM coating for rat calvarial bone regeneration.

Following micro-CT analysis, the fixed samples were decalcified in 10% EDTA for 2 months, dehydrated in 70%, 80%, 90% and 100% ethanol and embedded in paraffin. The prepared samples were sectioned into thick sections. Thereafter, the sections were stained with haematoxylin-eosin (H&E) and Masson’s trichrome to observe new bone formation and collagen deposition. Figure 1

### Statistical Analysis

All the data in this study were analyzed by origin and are expressed as the mean ± standard deviation. All the analyses were evaluated by analysis of variance (ANOVA). A *p* < 0.05 was regarded as statistical significance (*p < 0.05, **p < 0.01, ***p < 0.001).

## Results and Discussion

### Physicochemical Characterization

**FIGURE 2:**
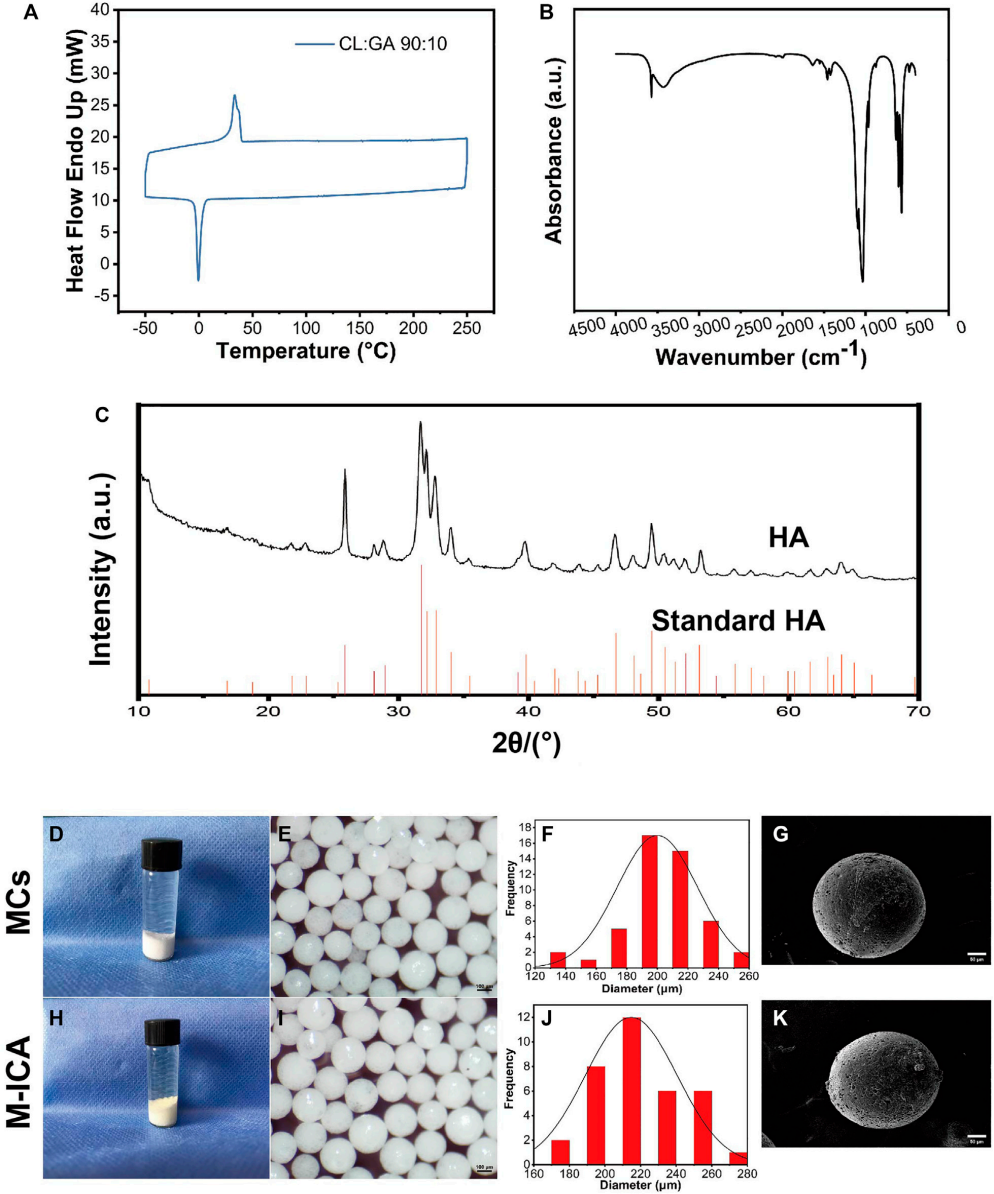
**(A)** DSC thermogram of PGCL. **(B)** FTIR spectra of HA. **(C)** XRD pattern of HA. **(D–K)** Gross observation **(D,H)**, macroscopic images **(E,I)**, size distribution **(F,J)**, SEM images **(G,K)** for MCs and M-ICA (Scale bar = 100 μm for e and i, 50 μm for g and k).

The PGCL polymers were characterized by GPC and differential scanning calorimetry (DSC). GPC results showed that the polymer had a Mn of 200,000 and a Mw of 130,000 with PDI of 1.5 (Table 2). The melting points (Tm) and crystallization (Tc) were 45.17°C and −0.87°C (Figure 2A), respectively. The above results confirmed that we successfully prepared PGCL polymer.

**TABLE 2:** GPC results of PGCL.

Weight ratio in feed CL/GA	M_n_ (g/mol)	M_w_ (g/mol)	PDI
PGCL 90:10	1.3 × 10^5^	2.0 × 10^5^	1.5

For HA, Figure 2B showed FTIR spectra of HA, the IR spectrum exhibited a characteristic broad absorption band at 3,570 cm^−1^ for the hydroxyl group. Meanwhile, phosphate absorption bands appeared at 1,075, 1,033, 940, 601, and 562 cm^−1^ which are all characteristic for a typical hydroxyapatite FTIR spectrum (Zhang et al., 2015). It indicated that we have successfully synthesized HA. Figure 2C showed the XRD patterns of HA and the standard data for the hexagonal hydroxyapatite. The results showed that the crystal plane spacing (d values) of HA was consistent with the standard HA spectra, indicating that the synthesized HA had standard crystal structures.

### Morphology of MCs and M-ICA

The morphology of microcarriers were shown in Figures 2D–K. On the whole, the surface of the MCs group showed a white color while the M-ICA group appeared a light-yellow color due to the loaded ICA. It indicated that ICA was successfully incorporated into the M-ICA microcarriers.

Both group of the microcarriers exhibited good sphericity with particle size in the range of 100–300 μm. To provide a homogeneous environment for cell growth, we screened microcarriers with particle size in the range of 200–300 μm. The particle sizes range of the microcarriers in groups MCs and M-ICA were close to each other, indicating that the loading of drug had no effect on the particle size of the microcarriers. The surface state and morphology of microcarriers were observed by SEM. It could be found that groups MCs and M-ICA both showed fine pores on the surface of microcarriers. This was caused by the volatilization of chloroform during the preparation of microcarriers.

### *In Vitro* Icariin Release Profile

**FIGURE 3:**
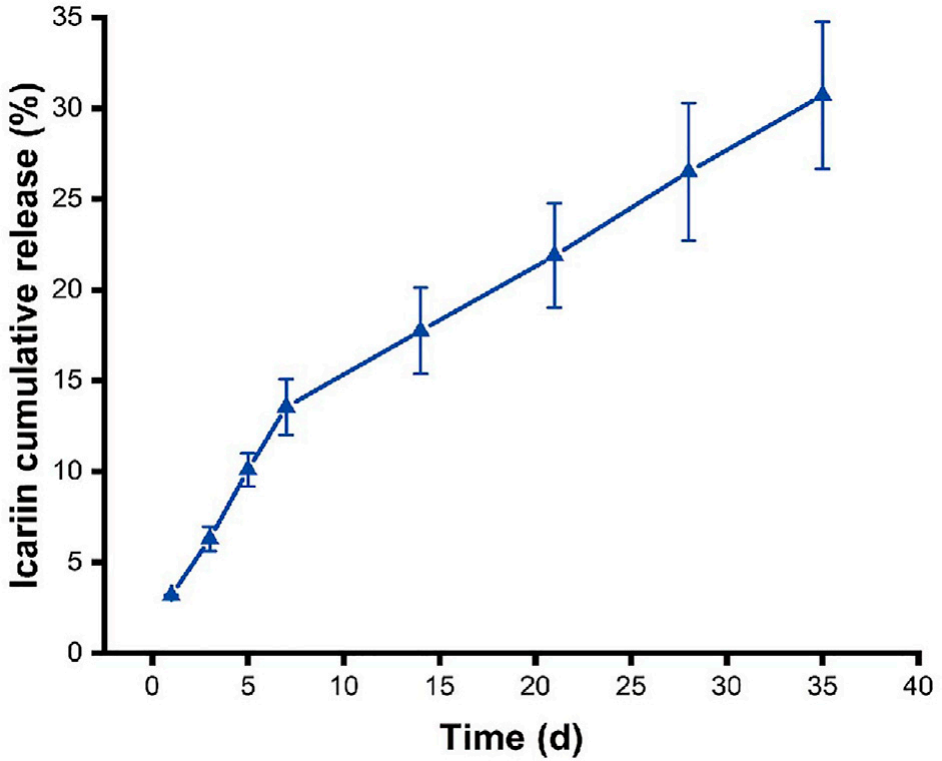
ICA cumulative release profiles for 1, 3, 5, 7, 14, 21, 28, and 35 days (*n* = 3).

ICA release behavior of the microcarriers was shown in Figure 3. It exhibited an initial burst release of ICA within the first 7 days, which was determined to be 13.6%. Then the drug showed sustained release and the cumulative release of ICA reached 30.7% by day 35 of incubation and was still continuing to be released. The drug release kinetics from biodegradable carriers is closely related to the degradation behavior of the materials (Wei et al., 2004). Ica behaved an initial burst release within the first 7 days due to the ICA being on the surface layer of microcarriers and gelatin dissolution driven drug release. After the initial burst release, the drug enters a slow release phase, which is due to diffusion and degradation of the polymer.

### Bio-Fabrication of Extracellular Matrix Coatings by Bone Marrow Mesenchymal Stem Cells

**FIGURE 4:**
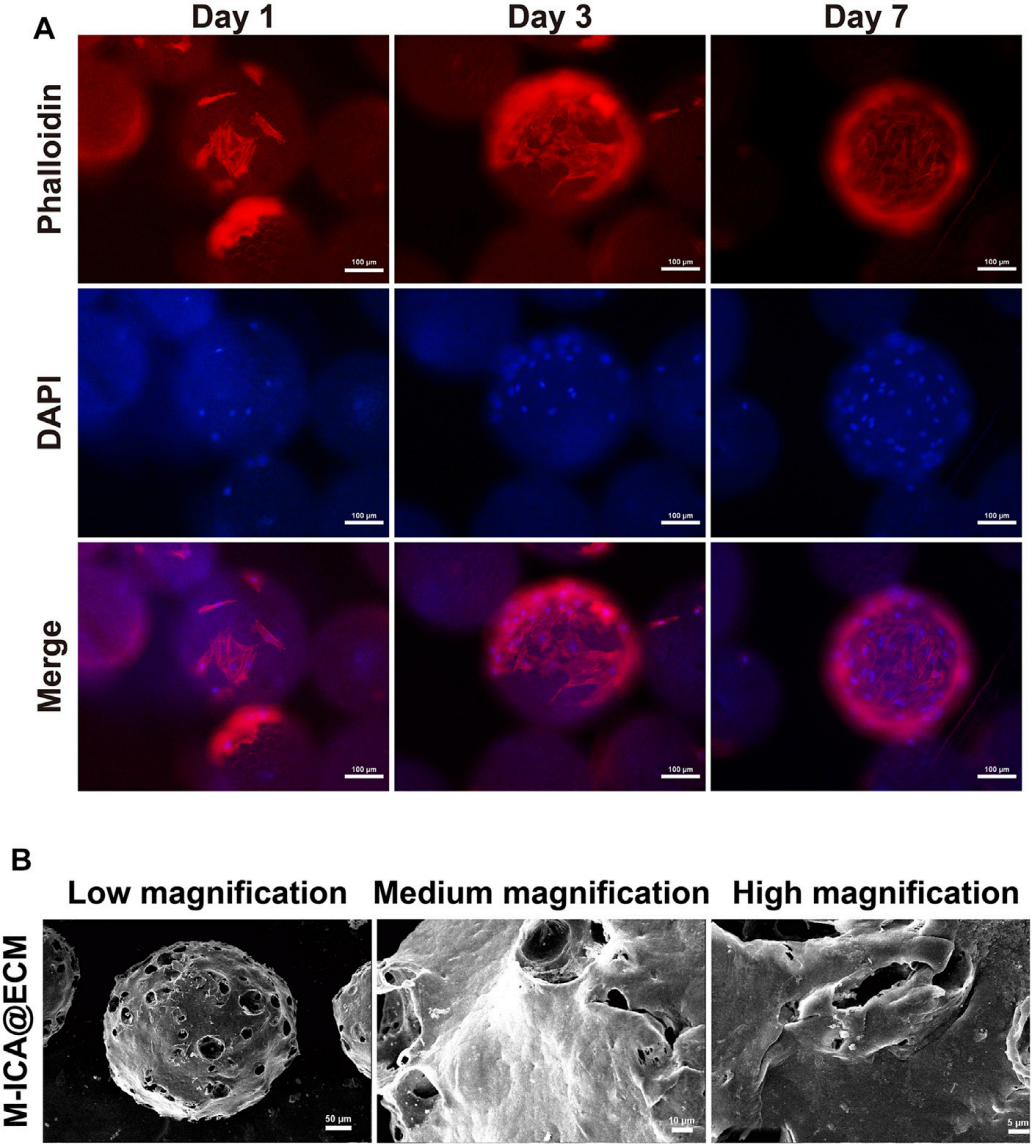
**(A)** Morphology of adhered BMSCs on M-ICA for 1, 3, and 7 days observed *via* rhodamine—phalloidin (actin microfilaments, red)/DAPI (nuclei, blue) staining (scale bar = 100 μm). **(B)** The morphology of M-ICA@ECM microcarrier observed under SEM (scale bar = 50 μm for low, 10 μm for medium, 5 μm for high magnification).

At 1, 3, and 7 days of culture, cell adhesion and spreading on the microcarrier were observed through fluorescence staining (rhodamine phalloidin and DAPI). As shown in Figure 4A, cells firmly adhered to the microspheres with good morphology. As time progressed, the number of attached cells on the microcarrier increased. After 7 days of culture, the cells almost completely covered the microspheres, and the cells on the microspheres grew densely and spread well. In addition, the cell surface at this time was coated with dense ECM. As a widely used material for tissue engineering scaffolds, gelatin has good biocompatibility and the function of supporting cell adhesion (Ouyang et al., 2017). Porosity should also facilitate the transfer of nutrients (Cui et al., 2018; Orapiriyakul et al., 2018; Grande Tovar et al., 2019). In this study, microcarriers containing PGCL, HA and gelatin exhibited excellent biocompatibility for supporting cell growth and proliferation. Based on these properties, the microcarriers we prepared could produce ECM by supporting cell adhesion and proliferation. In addition, gelatin could be gradually dissolved to produce pores on the surface of microspheres under physiological conditions, which we named “*in situ* pore production”. Such a design could prevent the loss of the drug in the microcarriers under nonphysiological conditions. At the same time, the porous microcarriers also provided a larger surface area for cell growth and drug release.

After culturing for 7 days, the microcarriers were processed immediately for decellularization. The decellularized microcarriers were observed by SEM. As shown in Figure 4B, the surface of the M-ICA@ECM microcarriers was almost completely covered with a cream-like ECM structure. In addition, the surface of the microcarriers showed a distinct porous structure due to the dissolution of gelatin. The purpose of decellularization is eliminate cells and cellular remnants to avoid from causing an immune response, while preserving ECM-derived proteins. The methods currently being used to decellularize can be divided into three main categories: physical, chemical, and biological (Crapo et al., 2011; Fernandez-Perez and Ahearne, 2019). In this study, we used physical decellularization to remove BMSCs from the surface of microcarriers. This method is easy to be performed and could avoid the destruction of ECM components by chemical reagents (Keane et al., 2015; Fernandez-Perez and Ahearne, 2019). Other studies have also shown that the method could significantly reduce antigenicity and highly preserve the ECM components (Deng et al., 2018; Deng et al., 2020; Dong et al., 2020).

### Osteogenic Extracellular Matrix Secretion Regulated by Icariin

**FIGURE 5:**
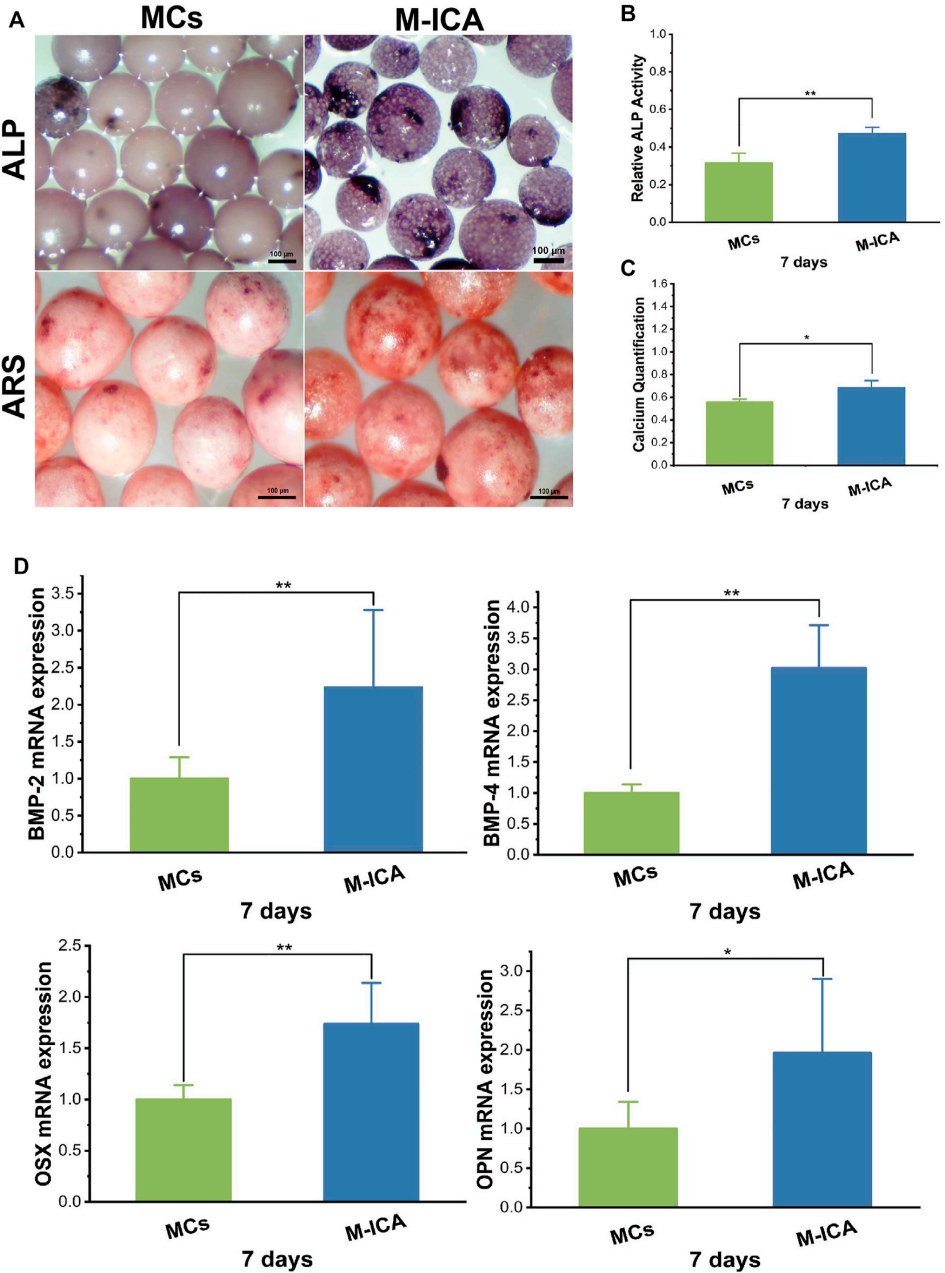
**(A)** ALP and ARS staining of BMSCs cultured for 7 days on MCs and M-ICA (Scale bar = 100 μm). The corresponding ALP quantitation **(B)** and corresponding calcium deposition quantitation **(C)** of BMSCs cultured for 7 days on MCs and M-ICA. **(D)** qRT-PCR analysis of BMP-2, BMP-4, OSX and OPN (*p < 0.05, **p < 0.01, *n* = 3).

Cell-derived ECM can recapitulate the natural bony ECM microenvironment. Some studies have reported that osteogenic ECM secreted by cells cultured under osteogenic induction conditions could significantly enhance mineralization deposition of osteoblasts(Datta et al., 2006). ICA could promote osteogenic differentiation of rat BMSCs (Jing et al., 2018) and ICA loaded in microcarriers could induce ECM-secreting cells to form osteogenic ECM during ECM formation. Therefore, we performed ALP and ARS assay to evaluation the effect of ICA on the osteogenic differentiation of BMSCs. As shown in Figure 5A, the results of ALP staining were observed under a stereomicroscope and the M-ICA group appeared deeper than the MCs group. Meanwhile, relative quantitation of ALP showed similar results (Figure 5B). Moreover, to investigate the effect of ICA on BMSC mineralization, ARS and calcium quantification were performed to characterize the mineralized nodules. ARS staining images were observed under stereomicroscope (Figure 5A). After 7 days of culture, more obvious alizarin red nodules appeared in the M-ICA group. The quantitation of mineral deposition showed similar results (Figure 5C). Based on these results, ICA provided osteoinductive culture conditions for ECM and promoted the formation of osteogenic ECM.

Also, we detected the expression of typical osteogenesis related genes after 7 days of culture. As shown in Figure 5D, the expression levels of BMP-2, BMP-4, OSX and OPN were all up-regulated in the M-ICA group compared with the MCs group. During the osteogenesis process, many bone-related genes and proteins will be activated (Tang et al., 2018). Bone morphogenetic proteins (BMPs) are the most important growth factors in bone regeneration. BMP-2 and BMP-4 both have strong osteoinductive ability (Liu et al., 2016; Waqas et al., 2019; Liang et al., 2020). It has been shown that ICA could up-regulate the expression of BMP-2 and BMP-4 in osteoblasts and significantly upregulate OSX at low doses (Yang et al., 2019). In this study, ICA regulated osteogenic ECM by inducing osteogenic differentiation of BMSCs during ECM formation. In addition, it has been demonstrated that growth factors such as BMP-2 could be retained in decellularized ECM (Onishi et al., 2018).

### Icariin Incorporation and Extracellular Matrix Coating Promoted the Migration of Bone Marrow Mesenchymal Stem Cells *in Vitro*

**FIGURE 6:**
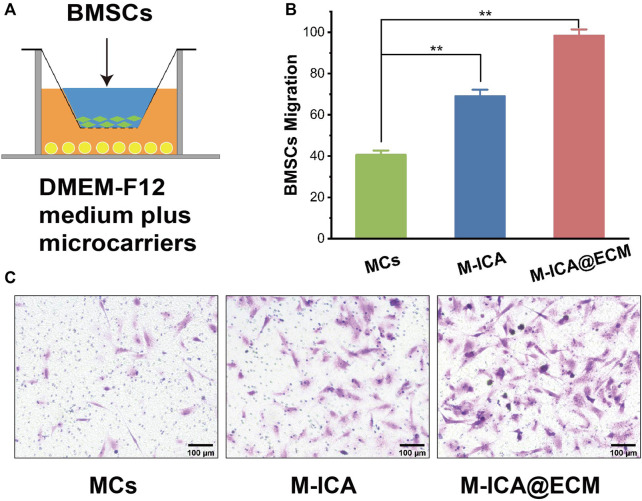
Transwell migration assay: **(A)** Schematic diagram of the transwell system; **(B)** Migrated BMSCs counted in different groups; **(C)** Microscope images of migrated BMSCs induced by MCs, M-ICA and M-ICA@ECM (Scale bar = 100 μm) (*p < 0.05, **p < 0.01, *n* = 5).

*In vitro* migration of BMSC were shown in Figure 6. It indicated that the number of migrating BMSCs in the M-ICA@ECM group was significantly higher than that in the MCs and M-ICA group. In addition, it was interesting to note that the number of migrating BMSCs in the M-ICA group was significantly higher than that in the MCs group.

BMSCs have the advantages of easy availability, abundant source, multidirectional differentiation potential, and can produce active ingredients to enhance wound healing, making them widely be used in tissue engineering. However, the low recruitment of BMSC in the tissue limited the repair effect (Jiao et al., 2018). Studies indicated that the bioactive substances derived from ECM could recruit large numbers of endogenous progenitor or stem cells to the site of injury for repairing tissue damage (Daley and Yamada, 2013; Wang et al., 2020). However, there is no fully unified answer of the migration mechanism. Deng et al. (2018) found that IGFBP3 was an important MSC homing molecule and IGFBP3 could promoted hBMSC migration. Dong et al. (2020) found that CXCL 12 played a crucial role in recruiting host cells. So it was thought that IGFBP3 and CXCL 12 might be contained in ECM for inducing cell migration. In recent years, some studies have shown that plant-derived components of ICA could promote the migration of BMSCs into damaged tissues and enhance the healing of damaged tissues (Cui et al., 2017; Maeda, 2020). Although the mechanism of BMSCs migration have not been clearly defined, Zhu et al. (2018) concluded that ICA promoted the migration of BMSCs by activating HIF-1α and further regulated the expression of CXCR4. It was observed that ICA significantly promoted the migration of BMSC by *in vitro* cell migration assay. So ICA and ECM coating may synergistically promote cell migration.

### Osteogenic Differentiation Evaluation *in vitro*

**FIGURE 7:**
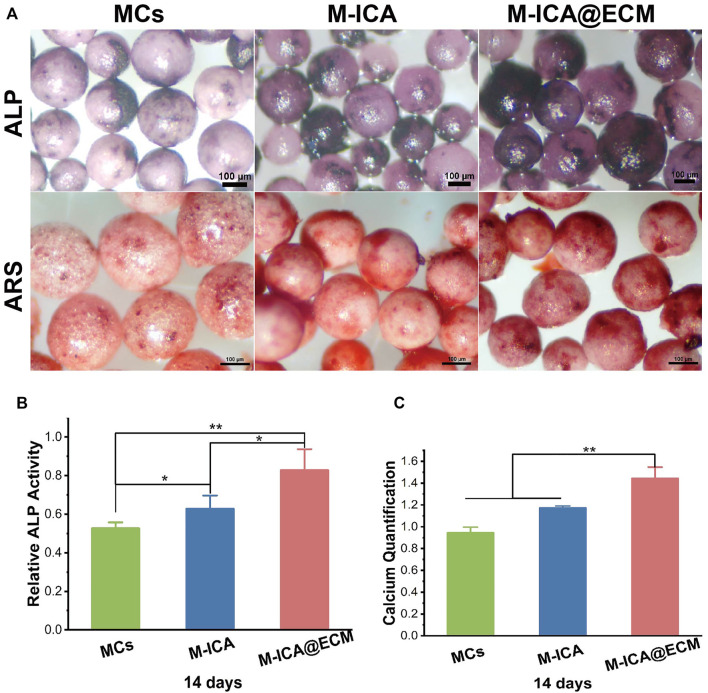
**(A)** ALP and ARS staining after incubation for 14 days on MCs, M-ICA, M-ICA@ECM (Scale bar = 100 μm). The corresponding ALP quantitative evaluation analysis **(B)** and corresponding calcium deposition quantitation **(C)** after cultured on MCs, M-ICA, M-ICA@ECM for 14 days. (*p < 0.05, **p < 0.01, *n* = 3).

The results of ALP and ARS staining and the ALP relative activity of BMSCs cultured on the different various components of microcarriers were shown in Figure 7A. BMSCs cultured on the M-ICA@ECM showed the deepest violet color in staining. Moreover, a deeper violet staining color was shown on the group M-ICA compared to group MCs. The ARS staining images also indicated that calcium deposition was greater on group M-ICA than MCs. Especially, group M-ICA@ECM exhibited the largest nodules. The ALP relative activity assay (Figure 7B) and the quantitative of mineral deposition (Figure 7C) were consistent to ALP staining and ARS staining. The results revealed that the additive effect of ECM coated microcarriers on early osteogenesis.

**FIGURE 8:**
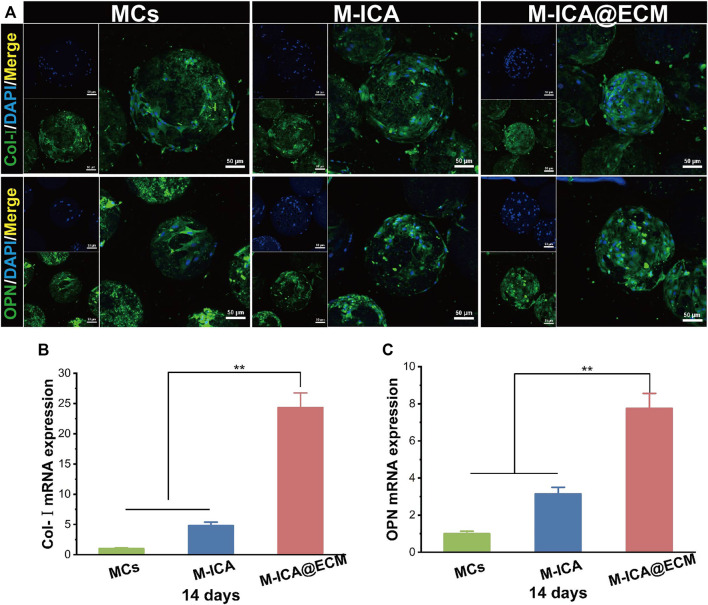
The expression of osteogenesis-related genes and proteins: **(A)** Immunofluorescent images of Col-I and OPN expressed by BMSCs cultured on the different microcarriers for 14 days which were observed under CLSM (Scale bar = 50 μm); **(B,C)** qRT-PCR analysis of Col-I and OPN. (*p < 0.05, **p < 0.01, *n* = 3).

To further confirm the above results, we evaluated the mRNA expression levels of OPN and Col-I by qRT-PCR (Figures 8B–C). The results showed that after 14 days of culture, the M-ICA@ECM group had the highest OPN and Col-I expression levels. It indicated that ICA and dECM coating had a synergistically great positive effect on the osteogenic differentiation of BMSCs. In addition, the mRNA expression level in the M-ICA group was also significantly higher than that in the control group, once again demonstrating the effect of ICA on the promotion of osteogenic differentiation of BMSCs. Also, the expression of OPN and Col-I was observed by immunofluorescence staining at 14 days, as shown in Figure 8A. The results showed that the highest secretion of the OPN and Col-I proteins was observed in group M-ICA@ECM, while protein secretion in the group M-ICA was slightly higher than that in the group MCs. The protein staining results were consistent with the mRNA expression. *In vitro* results indicated that ICA and osteogenic ECM synergistically up-regulated the expression of osteogenic-related genes and thus promoted the differentiation of BMSCs toward osteogenesis.

### Bone Regeneration Evaluation *Via* Calvarial Defect Model

**FIGURE 9:**
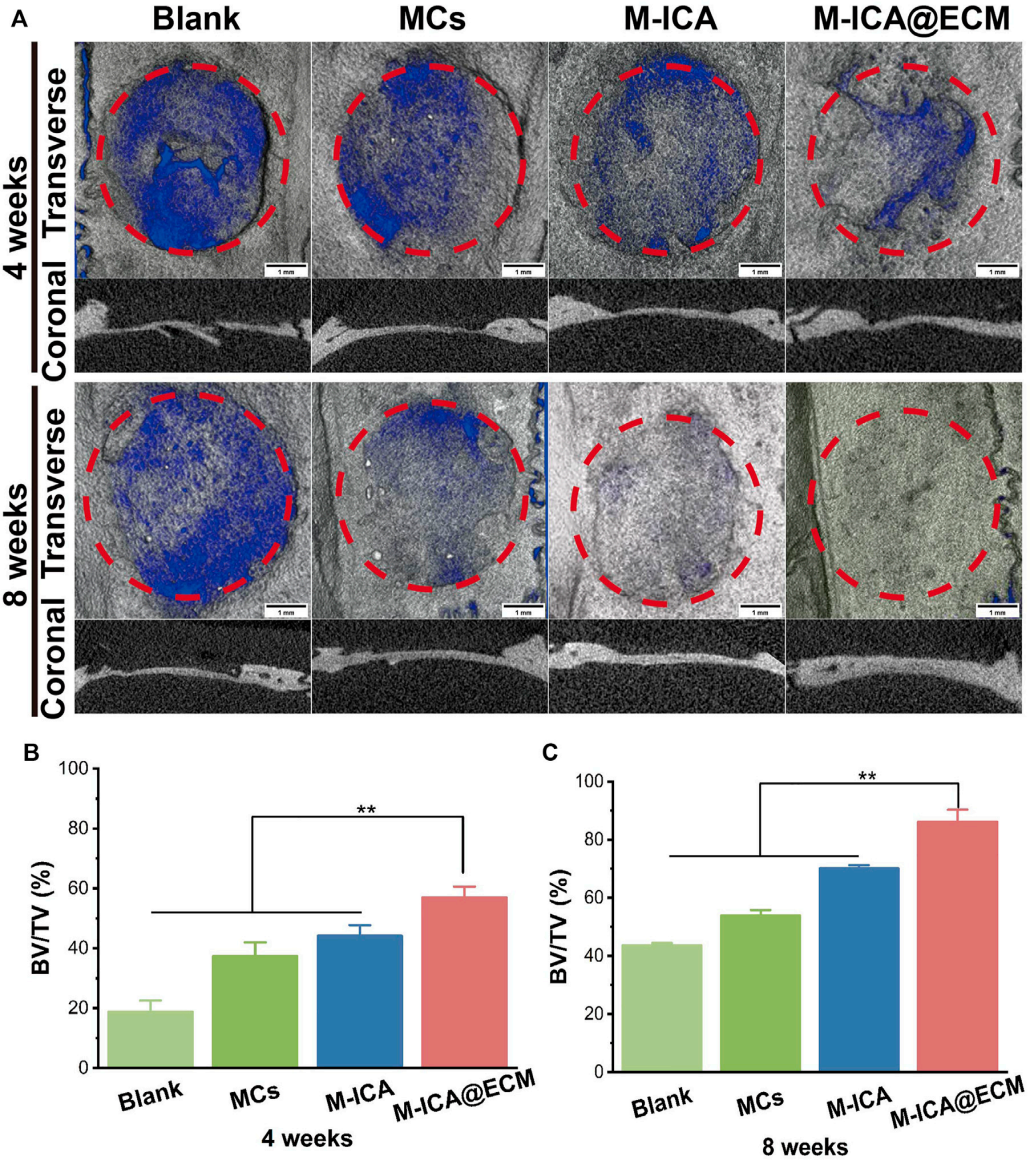
The rat calvarial bone repair after 4 and 8 weeks of implantation: **(A)** Micro-CT images of the rat calvarial bone (Scale bar = 1 mm) **(B,C)** The ratio of BV/TV at 4 and 8 weeks after implantation (*p < 0.05, **p < 0.01, *n* = 3).

Micro-CT was used to observe the formation of new bone 4 and 8 weeks after implantation. The 3D reconstructed images of different groups of calvarial defect were shown in Figure 9A. At 4 weeks, newly-formed bone was observed in the area surrounding the bone defect. The M-ICA@ECM group exhibited significantly better bone growth than the other three groups. After 8 weeks of implantation, a large number of defective areas were still visible in the blank group and MCs group, while very large new bone had been formed in the M-ICA and M-ICA@ECM groups. In particular, the defective area in the M-ICA@ECM group was almost completely repaired. The quantitative analysis of micro-CT was shown in Figures 9B, C. After 4 and 8 weeks of implantation, the bone volume/tissue volume (BV/TV) ratio in the M-ICA@ECM group was 56.99 ± 3.60 and 86.20 ± 4.12, respectively, which were significantly higher than that in the other three groups. The above results indicated that ICA had a positive effect on new bone formation *in vivo*. In addition, the osteogenic dECM coating presented an additional synergistic promotion capacity together with ICA for bone regeneration.

### Histological Analysis

**FIGURE 10:**
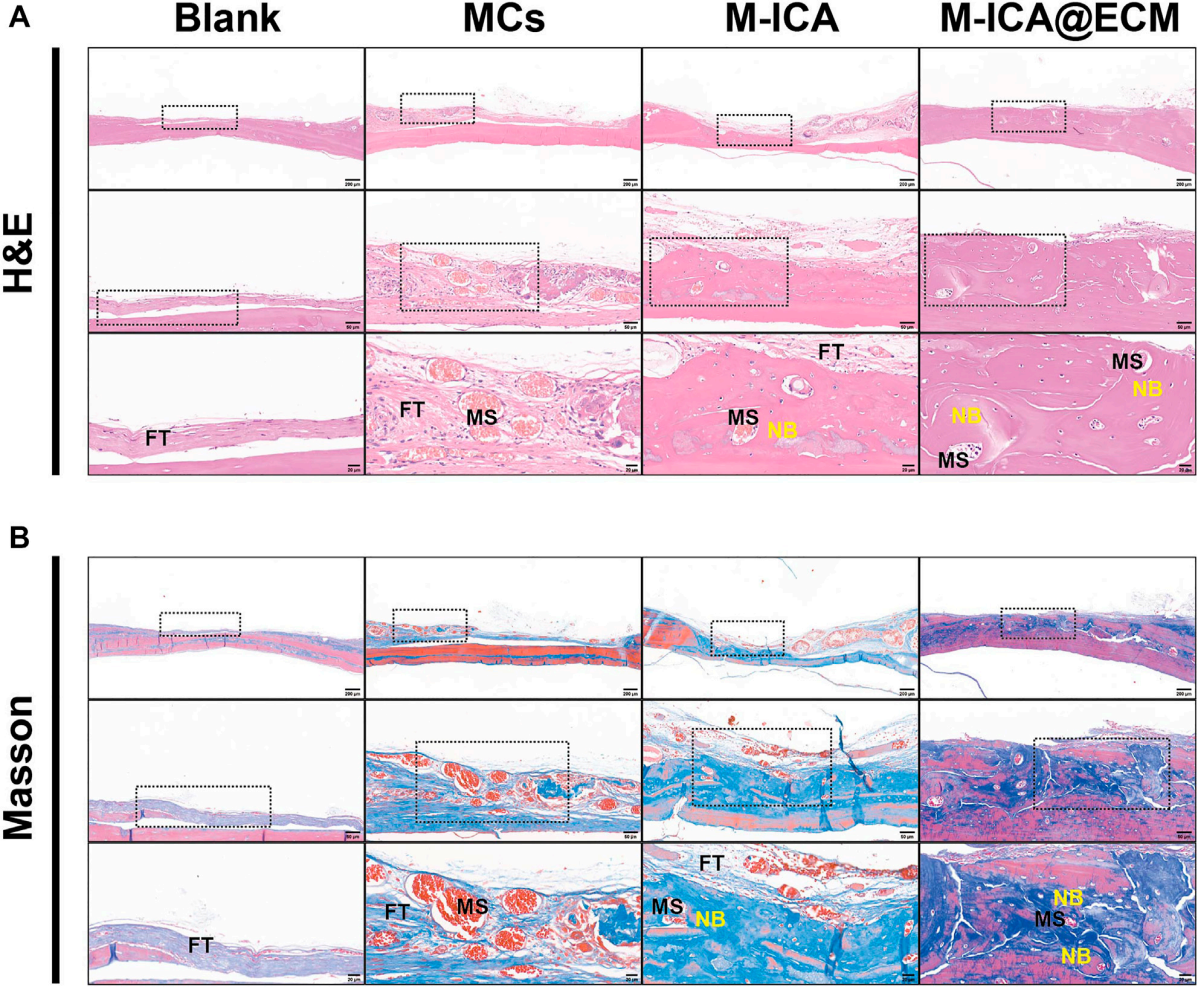
H&E staining **(A)** and Masson staining **(B)** in rat calvarial bone defects at 8 weeks after surgery. FT: fibrous tissue; MS: microcarriers; NB: new bone.

To further evaluate the bone tissue regeneration of the rat calvarial defects, H&E and Masson staining were performed. As shown in Figure 10, 8 weeks after implantation, there was only a small amount of fibrous connective tissue in the blank and MCs groups. In the M-ICA group, the microcarriers were surrounded by fibrous connective tissue A small amount of new bone was created at the same time. Moreover, the microcarriers were partially degraded and deformed, and the microcarriers were covered with newly formed blood vessels and connective tissue. While in the M-ICA@ECM group, a large amount of dense new bone tissue was formed, and the defect area was almost completely closed.

## Conclusion

The porous microcarriers prepared by the emulsionsolidification technique loading with ICA enhanced osteogenic activity compared with the normal PGCL microcarriers and regulated osteogenic ECM production during ECM formation. In addition, osteogenic dECM derived from BMSCs was successfully deposited on the surface of ICA-loaded porous microcarriers. The synergistic effect of dECM and ICA could further promote cell migration and osteogenic differentiation of BMSCs. Subsequently, M-ICA@ECM microcarriers also exhibited the best effects in repairing rat calvarial defects. In conclusion, the bionic porous microcarriers loaded with ICA and a dECM coating had both osteoconductivity and osteoinductivity properties, and they also had significant potential in the application of bone repair.

## Data Availability

The original contributions presented in the study are included in the article/Supplementary Materials, further inquiries can be directed to the corresponding authors.
