# Supplementary material for: Synergistically Promoting Bone Regeneration by Icariin-Incorporated Porous Microcarriers and Decellularized Extracellular Matrix Derived From Bone Marrow Mesenchymal Stem Cells
Source: Front Bioeng Biotechnol. 2022 Apr 7;10:824025. doi: 10.3389/fbioe.2022.824025 (PMC9021399; doi:10.3389/fbioe.2022.824025)
Supplement: Supplementary file 1 [file DataSheet1.docx]

Supplementary Material

# Morphological observation of BMSCs

After cell apposition, the cell morphology was observed using an optical microscope. The impurity cells in BMSCs almost disappeared after three generations of culture. The majority of BMSCs displayed long spindle-like morphology (**Supplementary Figure A-C**).

# Flow Cytometry Assay

BMSCs at passage 3 were harvested into flow tubes, and then washed three times with PBS. 1×10^5^ cells were incubated with fluorescently labeled antibodies CD29, CD90, CD45 for 30 min protected from **light**. The samples were washed with PBS and detected by flow cytometry. The results of flow cytometry confirmed that over 99.96% and 99.61% of BMSCs were positive for the CD29 and CD99 respectively. There were negative for CD45 (**Supplementary Figure D-F**).

# Osteogenic Induction Assay and Alizarin Red S Staining

Cells in the logarithmic **growth** **phase** **were** harvested and seeded in six-well plates at 2×10^4^ cells/cm^2^. The cells were cultured in BMSCs Osteogenic Differentiation Medium (Cyagen Biosciences Inc.), and the medium was changed every three days. After 2 weeks of culture, the cells were stained with ARS to evaluate the osteogenic differentiation ability. The results showed that the BMSCs appeared a large number of calcium nodules, indicating that the cells had good potential of osteogenic differentiation (**Supplementary Figure G-I**)

## Supplementary Figures


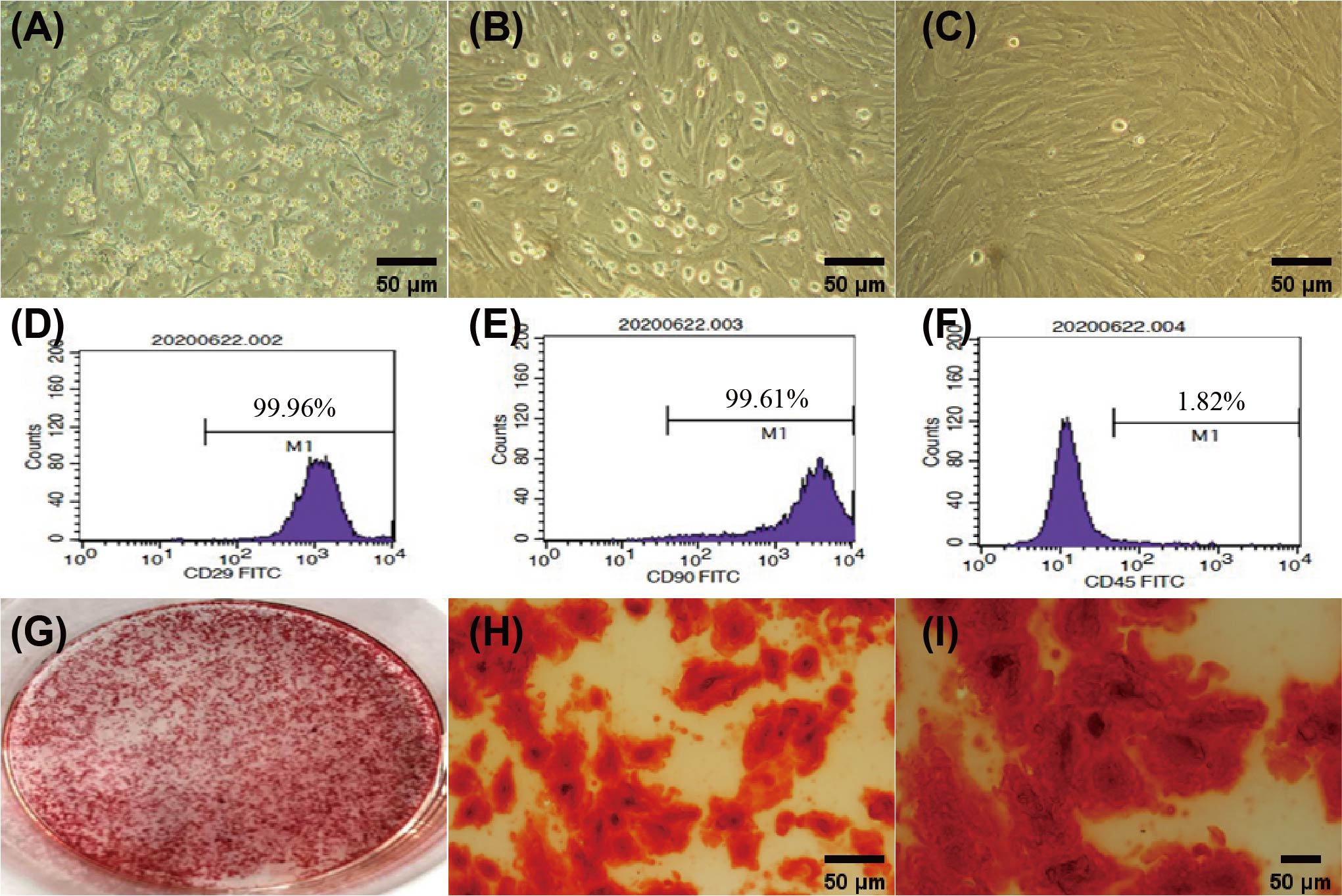


**Supplementary Figure 1.** BMSC identification (A-C) Micrographs of 1st , 2nd , 3rd isolated cells; (D-F) The expression of mesenchymal stem cell markers (CD29, CD90) and hematopoietic cell markers (CD45) for the isolated cells by flow cytometry; (G-I) Macroscopic and microscope images of isolated cells stained with ARS. (Scale bar = 50 μm).
